# Low maternal pertussis protection and quantified infant risk: supporting maternal Tdap vaccination in China

**DOI:** 10.3389/fimmu.2025.1687663

**Published:** 2026-01-02

**Authors:** Wen Wang, Tingting Zhang, Xiang Sun

**Affiliations:** 1 Department of Rheumatology and Immunology, Jiangsu Province (Suqian) Hospital, Suqian, Jiangsu, China; 2 Medical Record Office, Children’s Hospital of Nanjing Medical University, Nanjing, Jiangsu, China; 3 School of Public Health, Nanjing Medical University, Nanjing, Jiangsu, China; 4 Department of Expanded Program on Immunization, Jiangsu Provincial Center for Disease Control and Prevention, Nanjing, Jiangsu, China

**Keywords:** maternal, pertussis, risk modeling, seroepidemiology, Tdap vaccination

## Abstract

**Background:**

Pertussis remains a significant threat to infants, particularly in settings like China, where maternal tetanus-diphtheria-acellular pertussis (Tdap) vaccination is not part of the National Immunization Program, and data on maternal pertussis immunity are limited. This study aimed to assess the seroprevalence of protective pertussis toxin (PT) IgG antibodies in pregnant women and quantify the potential risk of infant pertussis under varying epidemiological scenarios.

**Methods:**

A cross-sectional serosurvey was conducted among 1,205 pregnant women in Jiangsu Province, China, from January to December 2024. Serum anti-PT IgG concentrations were measured using ELISA, with a protective threshold defined as ≥40 IU/mL. A scenario-based risk model (*P_infant_* =(1− *p_m_*)×*I_w_*×*E*×*T*) was developed to estimate infant pertussis risk (0–6 months) by linking maternal protection proportion (*p_m_*), age-specific incidence in childbearing-age women *I_w_*), exposure window (*E*), and mother-to-infant transmission probability (*T*).

**Results:**

The overall median anti-PT IgG concentration was 6.34 IU/mL (IQR: 4.24–11.16), with only 3.57% (95% CI: 2.66–4.77%) of women achieving the protective threshold (≥40 IU/mL). Under a representative scenario (*I_w_* = 50/100,000, *T* = 0.20), the model estimated 4.82 infant cases per 100,000 births under current immunity, reduced to 1.00 cases with maternal Tdap (*p_m_* = 80.0%), a 79.2% relative reduction. Higher incidence and transmission scenarios yielded greater absolute reductions with vaccination.

**Conclusion:**

Low maternal pertussis immunity in China poses a quantifiable risk to infants, particularly under elevated incidence scenarios. Maternal Tdap vaccination could substantially reduce infant pertussis burden, supporting its consideration for national immunization policy. These findings provide critical evidence for addressing immunity gaps and protecting vulnerable newborns in China.

## 1 Background

Pertussis, an acute respiratory infection caused by *Bordetella pertussis*, is highly contagious and transmitted via respiratory droplets. Clinically, it is characterized by paroxysmal cough, and in severe cases, can progress to apnea, pneumonia, or encephalopathy (1). Since the mid-20th century, widespread childhood vaccination programs have significantly reduced pertussis incidence; however, global surveillance over the past two decades has documented a resurgence, particularly in populations with waning immunity (2–4). The disease burden is even heavier in immunocompromised individuals, who face higher severity and complication rates. This resurgence highlights the urgent need for targeted immunization strategies to address immunity gaps across all age groups.

In China, the inclusion of pertussis-containing vaccines in the National Immunization Program has drastically lowered childhood incidence. Chinese children receive pertussis-containing vaccines (DTwP or DTaP), at 3, 4, and 5 months of age, with a booster at 18 months; coverage rates for the primary series exceed 95% nationally (5). Despite high coverage rates for the primary childhood series, immunity wanes over time, leading to low seroprevalence in childbearing-age women. Literature indicates that this observed waning is largely driven by the inherent immunological properties of the vaccines rather than solely by low uptake. Systematic reviews and meta-analyses show that acellular pertussis vaccines (aP/DTaP/Tdap) induce less durable protective immunity than whole-cell vaccines (wP/DTwP); however, the widespread use of wP vaccines has been constrained by their higher reactogenicity (6–8). Consequently, the limited durability of current acellular vaccines poses a challenge to sustaining long-term herd immunity. This is reflected in recent national surveillance data, which show a fluctuating upward trend in reported cases (5). Although the incidence in women aged 20–35 years remains lower than in children, it is not negligible, and some regional data suggest a gradual increase in this age group (9, 10). Given their close contact with newborns, susceptibility in this population serves as a key source of infant exposure. This immunity gap, driven by vaccine characteristics and evidenced by epidemiological trends, underscores the necessity of targeted strategies such as maternal Tdap vaccination to rapidly restore protection for both mothers and infants.

Newborns are at particularly high risk of severe pertussis due to incomplete primary immunization in the first months of life, with a case fatality rate exceeding that of any other age group (11). Passive transplacental transfer of maternal immunoglobulin G (IgG) antibodies is the primary source of early-life protection (12). Elevating maternal antibody levels during pregnancy has been shown to reduce mother-to-infant transmission and significantly decrease infant pertussis morbidity and mortality. Consequently, the World Health Organization (WHO) recommends routine administration of a pertussis-containing vaccine (e.g., tetanus–diphtheria–acellular pertussis, Tdap) during pregnancy. Evidence from the United States (13) and United Kingdom (14, 15) that antenatal Tdap vaccination substantially increases pertussis antibody levels in both mothers and newborns, while markedly reducing infant pertussis incidence and deaths.

In China, Tdap vaccination has not yet been integrated into the National Immunization Program, and systematic data on maternal pertussis immunity remain scarce (16). Furthermore, no prior studies have quantitatively linked maternal antibody levels to age-specific pertussis incidence to estimate infant infection risk via maternal transmission. To fill this gap, we conducted the first seroepidemiological survey of pertussis toxin (PT) IgG levels among pregnant women in China, combined with national incidence data for women of childbearing age and infants. We developed a scenario-based risk model that links “maternal antibody protection → incidence among childbearing-age women → probability of mother-to-infant transmission,” enabling quantitative estimation of infant pertussis risk under varying epidemiological contexts.

By integrating empirical antibody prevalence with incidence-based transmission scenarios, our study quantifies the potential impact of maternal immunity gaps on infant disease risk. The findings provide direct evidence to inform the potential public health benefits of introducing routine antenatal Tdap vaccination in China, thereby supporting policy decisions aimed at reducing the newborn pertussis burden and improving maternal child health outcomes.

## 2 Methods

### 2.1 Study population and data source

We conducted a cross-sectional seroepidemiological study involving 1,205 pregnant women who attended routine antenatal care at sentinel hospitals in Jiangsu Province, China, from January to December 2024. Exclusion criteria included age <18 years, recent laboratory-confirmed chronic infectious diseases, immune-related disorders, or other chronic conditions (e.g., diabetes, hypertension, hepatic or renal disease). Eligible participants were healthy pregnant women with age ≥18 years and no history of pertussis infection or vaccination during the current pregnancy, all of whom provided written informed consent. The study protocol was approved by the [The Ethics Committee of Jiangsu Provincial Center for Disease Control and Prevention, approval No. JSJK2023-B027-02]. At enrollment, demographic characteristics—including age and gestational week—were recorded. National pertussis incidence data for women of reproductive age were extracted from surveillance records and modeled under both routine passive surveillance and higher-transmission scenarios (see Risk modeling).

### 2.2 Laboratory testing

Maternal serum anti-PT IgG concentrations were quantified by ELISA test kit (Virion\Serion, Germany) and expressed in IU/mL standardized to the WHO International Standard (NIBSC 06/140). All testing was performed at the central laboratory of the Affiliated Suqian First People’s Hospital of Nanjing Medical University. We used conventional interpretive bands: The <5 IU/mL level aligns with the assay’s limit of detection, indicating seronegativity; 5–<40 IU/mL reflects low or waning immunity from remote exposure; and ≥40 IU/mL serves as a putative protective threshold, correlating with sufficient antibody levels for potential transplacental protection in maternal studies (17).

### 2.3 Risk modeling

Maternal passive protection was pre-specified as PT IgG ≥40 IU/mL. To quantify the potential pertussis risk in infants aged 0–6 months in the absence of routine maternal Tdap, we constructed a simple scenario-based probability model (18–20):

*P_infant_* =(1− *p_m_*)×*I_w_*×*E*×*T*

- *P_infant_*= probability of infant pertussis in the first 6 months of life
- *p_m_* = proportion of pregnant women with PT IgG ≥ 40 IU/mL (maternal protection)
- *I_w_* = annual pertussis incidence among women of childbearing age (per person per year)
- *E*= exposure window fraction (0.5, representing 6 months)
- *T*= conditional mother-to-infant transmission probability upon maternal infection

We evaluated *I_w_* at five scenarios representing passive-to-elevated epidemiologic contexts in China: 2.15, 10, 50, 100, and 500 per 100,000 person-years. For *T*, we adopted a literature-informed range 0.05–0.30, consistent with household/close-contact pertussis transmission probabilities to infants (21, 22). In secondary “vaccinated” counterfactuals, we assumed a maternal protection level *p_m_* = 80.0% (In this study, the maternal protection level (pm) is used as a model parameter to estimate the potential reduction in infant pertussis risk following maternal Tdap vaccination. The baseline maternal protection level (pm) in our cohort was empirically measured as 3.57%, based on the proportion of women with IgG levels ≥40 IU/mL. For the counterfactual scenario of a high-coverage maternal Tdap program, we used *p_m_* = 80.0%. This value represents a realistically achievable level of maternal protection based on available evidence from studies of maternal Tdap vaccination programs in other countries. These studies demonstrate that after maternal Tdap vaccination, a significant proportion of women (more than 90.0%) achieve IgG levels above 40 IU/mL, providing substantial protection for their infants. This 80.0% level reflects the potential coverage and immune response rates observed in these real-world programs, rather than being an arbitrary threshold or experimentally derived value) (23, 24).

We report *P_infant_* and its translation into expected infant cases per 100,000 births for interpretability: *Cases_0–6m_*  =  100,000×*P_infant_*

### 2.4 Statistical analysis

Geometric mean concentrations (GMCs) were summarized as medians with interquartile ranges (IQRs), and categorical variables as counts with percentages. The maternal protection proportion *p_m_* (defined as PT IgG ≥40 IU/mL) was estimated with 95% confidence intervals. Between-group comparisons were performed using the *χ²* test (or the Cochran–Armitage trend χ² test where applicable) for categorical variables, and the Mann–Whitney U test (or Kruskal–Wallis H test for comparisons across more than two groups) for continuous variables. Scenario-based risk calculations were conducted on grids of *I_w_* and *T*; results were presented in tables, bar charts (fixed *T* = 0.20), and heatmaps. All analyses were conducted using R and Python. Two-sided *p* < 0.05 was considered statistically significant.

## 3 Result

### 3.1 Maternal serology and protection proportion

**Figure 1:**
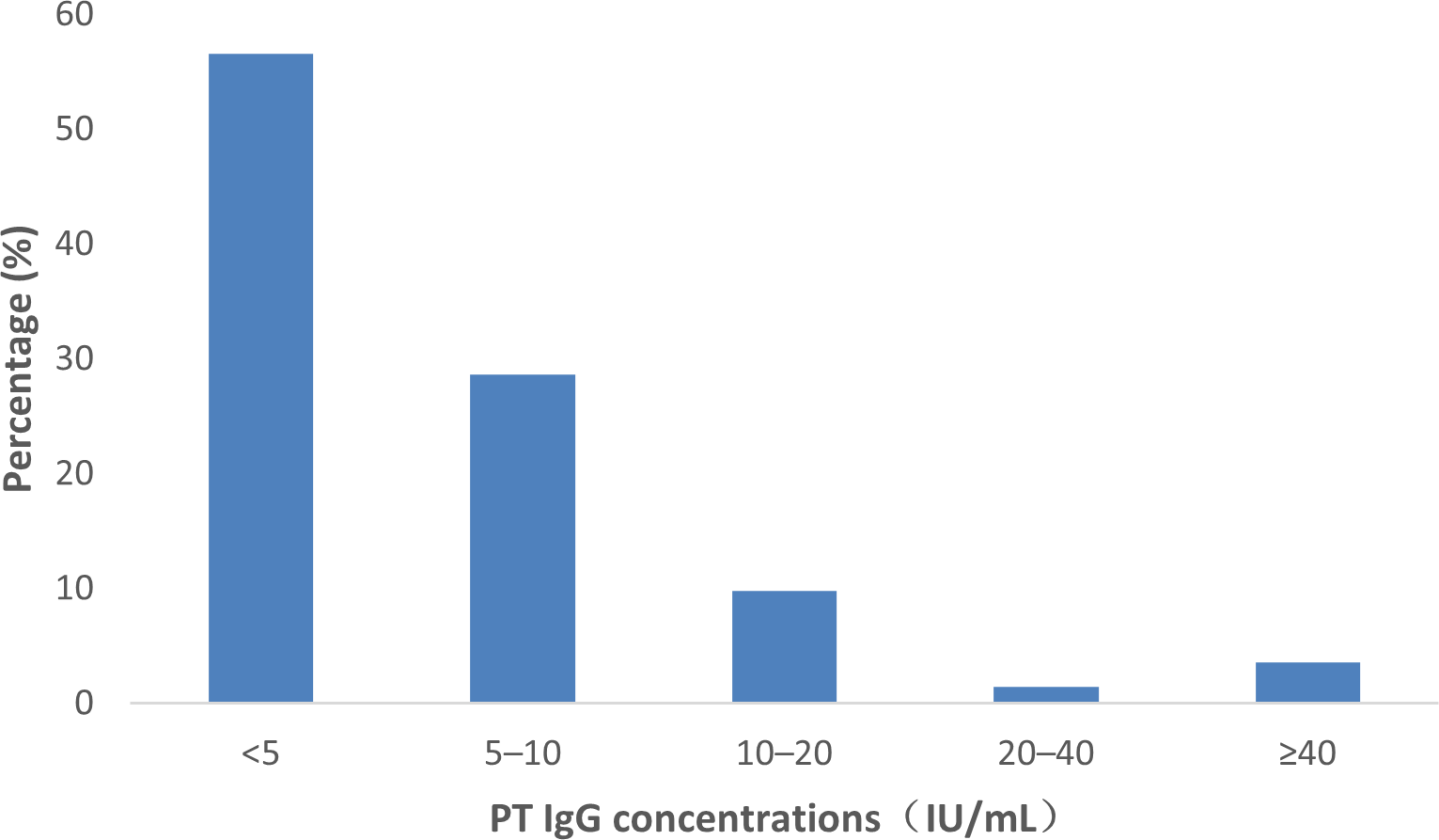
Distribution of maternal anti-pertussis toxin (PT) IgG concentrations.

A total of 1,205 pregnant women with valid PT IgG quantification were included in the analysis. The overall median (IQRs) concentration was 6.34 IU/mL (4.24–11.16). When stratified by maternal age and gestational week, the proportion of women with PT IgG levels ≥40 IU/mL ranged from 1.64% to 4.88%, with no statistically significant differences observed between strata (p>0.05). Median (IQR) PT IgG levels were also comparable across subgroups, ranging from 5.99 (3.98–10.95) IU/mL in women aged 23–27 years to 7.15 (4.51–12.47) IU/mL in those at ≥38 gestational weeks (p>0.05). Based on the pre-specified threshold of ≥40 IU/mL, the maternal protection proportion was calculated as: *p*_m_=3.57 (2.66–4.77)%. (**Table 1**, **Figure 1**).

**Table 1:** Maternal anti- PT IgG levels and protection proportion by age and gestational week.

Variables	N	PT IgG ≥40 IU/mL (%,95% CI)	P value	PT IgG, GMC M(Q_1_-Q_3_) (IU/mL)	P value
Age(years)			0.754		0.194
18-22	123	4.88(2.26-10.24)		6.34(4.26-10.15)	
23-27	394	3.81(2.32-6.19)		5.99(3.98-10.95)	
28-32	401	3.49(2.09-5.77)		6.33(4.30-12.36)	
≥33	287	2.79(1.42-5.41)		6.89(4.55-11.67)	
gestational weeks			0.147		0.075
30-33	584	4.62(3.19-6.64)		6.54(4.45-11.03)	
34-37	560	2.68(1.63-4.37)		6.09(3.98-11.22)	
≥38	61	1.64(0.29-8.72)		7.15(4.51-12.47)	
Total	1205	3.57(2.66-4.77)		6.34(4.24-11.16)	

### 3.2 Scenario model outputs

**Figure 2:**
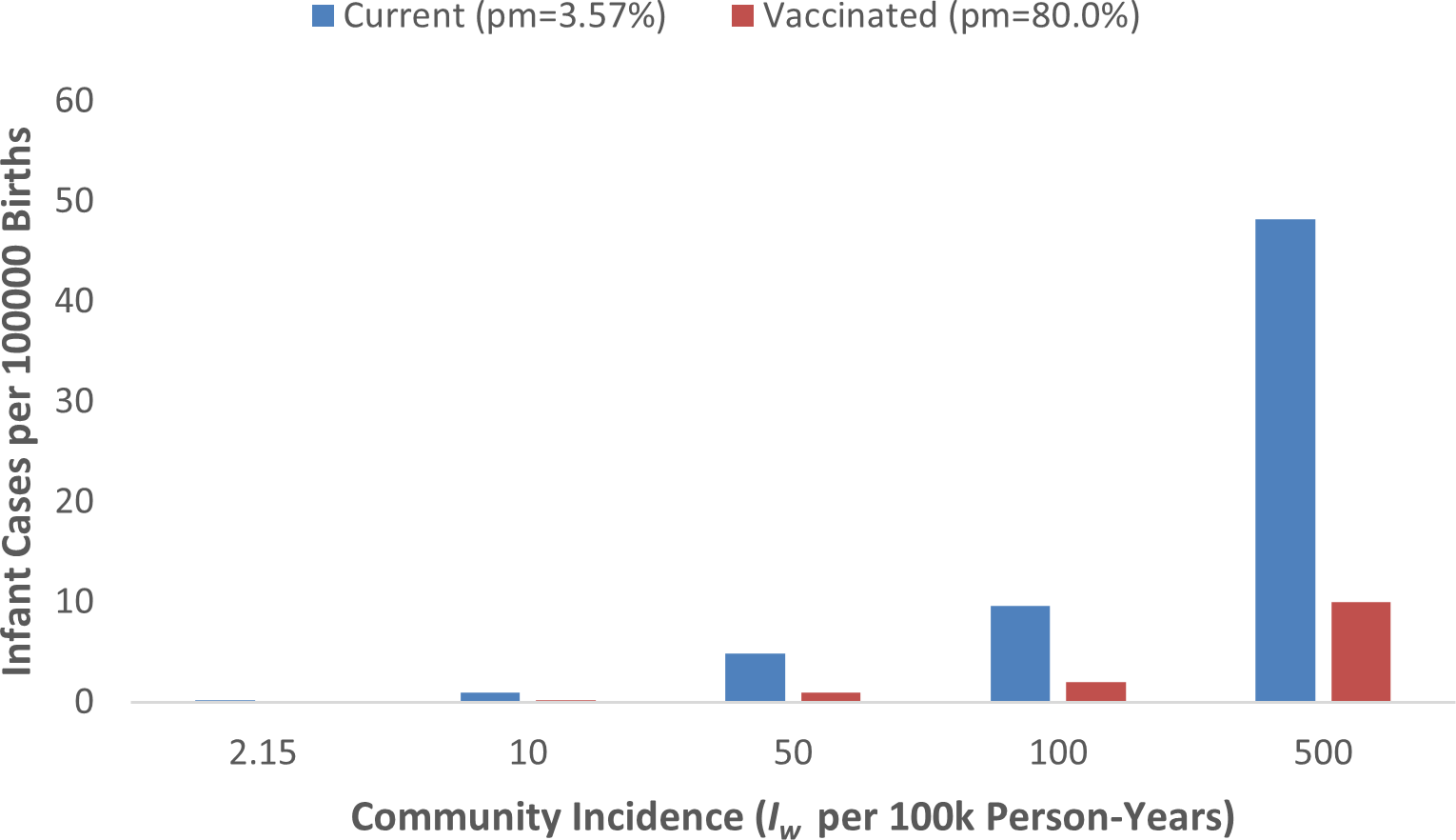
Projected infant pertussis cases (0–6months) per 100,000 births under current versus vaccinated maternal immunity (p_m_ = 80.0%, assuming T = 0.20 and E = 0.5).

Using the simplified risk model *P_infant_* =(1− *p_m_*)×*I_w_*×*E*×*T*, we explored a range of community maternal incidence values *I_w_* (2.15–500 cases per 100,000 per year) and mother to infant transmission probabilities *T* (0.05–0.30). For a representative scenario (*I_w_* = 50 per100,000·year, *T* = 0.20, exposure window *E* = 0.5), the current maternal protection level (*p_m_* = 0.0357 at ≥40 IU/mL) corresponds to an estimated 4.82 cases per 100,000 births among infants aged 0–6 months. If maternal protection were raised to *p_m_* = 0.80 (plausible after high-coverage maternal Tdap), the same scenario yields 1.00 cases per 100,000 births, an absolute reduction of 3.82 cases per 100,000 births (79.2% relative reduction) (**Figure 2**, **Table 2**).

**Table 2:** Infant pertussis cases per 100,000 births: current vs. vaccinated scenarios.

*I_w_* per 100k PY	Current cases/100k births	Vaccinated cases/100k births	Absolute reduction
2.15	0.207	0.043	0.164
10	0.964	0.200	0.764
50	4.822	1.000	3.822
100	9.643	2.000	7.643
500	48.216	10.000	38.216

### 3.3 Sensitivity analysis

**Figure 3:**
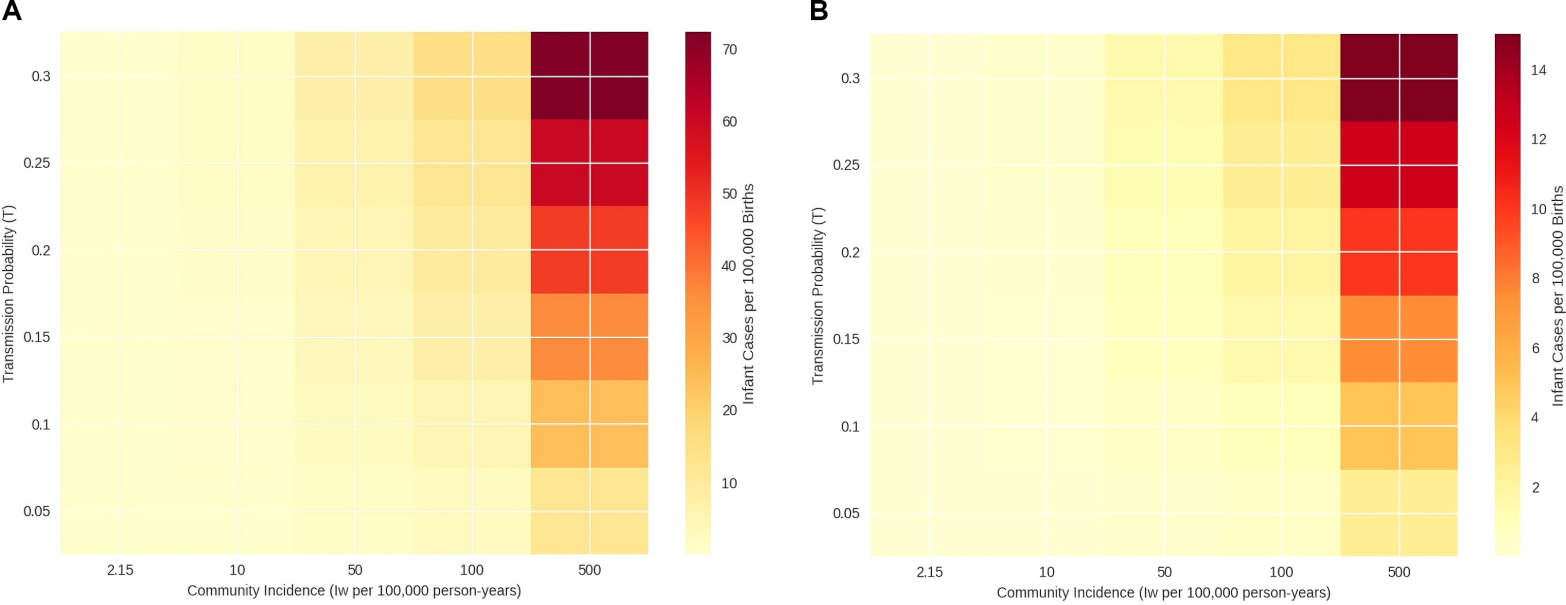
**(A)** Heatmap of expected infant pertussis cases per 100,000 births under current maternal protection (*p_m_* = 3.57%). **(B)** Heatmap of expected infant pertussis cases per 100,000 births under a vaccinated counterfactual (*p_m_* = 80.0%).

The sensitivity analysis demonstrates that the absolute reduction in infant pertussis cases increases with both community incidence (*I_w_*) and the maternal-to-infant transmission probability (*T*). In higher-incidence and higher-transmission scenarios (e.g., *I_w_* = 500 per 100,000; *T* = 0.30), the projected disease burden under current maternal immunity rises markedly (up to 72.3 cases per 100,000 births in our grid) (**Figure 3A**). Under these conditions, increasing maternal protection (pm→80.0%) yields a substantial absolute reduction in projected cases (**Figure 3B**).

## 4 Discussion

In this seroepidemiological study of 1,205 pregnant women, we found a low prevalence of putative protective PT IgG levels (≥40 IU/mL), with an overall maternal protection proportion *p*_m_ of 3.57% (95% CI 2.66–4.77%). When these serological results were combined with scenario-based incidence inputs and plausible mother-to-infant transmission probabilities, the modeled infant pertussis risk in the first 6 months was low under current low-incidence scenarios but rose substantially under assumptions of higher community incidence and transmission. Notably, maternal vaccination—modeled here as raising *p*_m_ to 0.80—produced a large absolute reduction in infant cases in those high-risk scenarios. These findings, which link a measurable maternal immunity gap to quantifiable infant risk, provide empirical support for considering antenatal pertussis immunization strategies in China.

Our results align with international evidence that maternal Tdap vaccination substantially reduces infant pertussis incidence and severe outcomes. In England, the introduction of a maternal pertussis vaccination program in 2012 was followed by observational and case–control evaluations reporting high vaccine effectiveness against pertussis in very young infants (for example, VE = 91%[95%CI 84–95%] and an adjusted VE = 93%[95%CI 81–97%]), as well as reductions in infant hospitalizations and deaths after program implementation. Similarly, in the United States-where maternal Tdap was recommended beginning in 2011-national surveillance analyses have documented a sustained decrease in pertussis incidence among infants <2 months of age in the Tdap era (13, 15, 25). These real-world, pre/post implementation data further support our modeling conclusion that maternal Tdap can substantially reduce the burden of pertussis in the most vulnerable early infancy period, particularly when community transmission is elevated.

Our findings of low maternal antibody levels in China should be interpreted in the context of broader evidence on pertussis vaccine durability. The literature indicates that the observed waning is substantially attributable to the shorter durability of immunity induced by acellular pertussis vaccines, rather than solely low uptake (6–8). Whole-cell vaccines tend to induce more durable responses, but their higher reactogenicity has limited their reintroduction in many settings. Policy implications are therefore twofold: in the short term, high-coverage maternal Tdap programs provide a practical and evidence-based means to rapidly enhance passive neonatal protection; in the longer term, investment in vaccine development (improved aP formulations, novel adjuvants, or alternative antigens) and evaluation of different immunization strategies is needed to achieve more durable population immunity and reduce reliance on frequent boosters. Ongoing surveillance and programmatic evaluation will be essential to balance immediate infant protection with long-term immunological goals.

From a public health and policy perspective, two points merit emphasis. First, in scenarios involving low incidence as reported by passive surveillance, the absolute number of infant cases attributed to maternal susceptibility may appear small (26). However, these cases are concentrated in highly vulnerable infants aged 0–6 months, who face relatively severe clinical outcomes (including hospitalization, severe illness, or even death). Thus, their public health significance should not be overlooked despite the small absolute numbers. Second, when community incidence rises or local outbreaks occur, our model indicates that the number of infant cases linked to inadequate maternal immunity will increase proportionally. In such high-risk contexts, the absolute benefits of introducing maternal Tdap vaccination during pregnancy are particularly pronounced (27, 28). Therefore, we recommend that when formulating vaccination strategies, reliance should not be placed solely on current passive surveillance data indicating low incidence; instead, consideration should be given to the potential underestimation of actual incidence and the risk-amplifying effects of potential outbreaks. International experience also shows that the public health returns of maternal immunization grow greater as community transmission increases. Furthermore, our findings have broader applicability to other countries without national maternal Tdap immunization programs, such as Bulgaria and Estonia in Europe, and Paraguay and Venezuela in Latin America, where low maternal immunity may similarly pose risks to infants, supporting the consideration of antenatal vaccination strategies in these settings (29, 30).

To further illustrate the potential national impact in China, we extrapolated our scenario-based estimates using the approximate 9.5 million annual births reported for 2024 (31). Under the representative scenario (*I_w_* = 50 per 100,000 PY, T = 0.20), maternal Tdap vaccination (increasing *p_m_* to 80.0%) could prevent approximately 364 infant pertussis cases (0–6 months) annually nationwide. In a higher-incidence scenario (*I_w_* = 500 per 100,000), this prevention potential rises to about 3,645 cases, highlighting the substantial public health benefits during outbreaks or resurgence periods (**Supplementary Table S1**).

In terms of program implementation, if maternal Tdap vaccination is introduced in China, key considerations should include: the timing of vaccination (most studies support administration during the second or third trimester to maximize placental antibody transfer); integration with existing prenatal care systems to ensure high coverage (32); and the establishment or strengthening of surveillance and evaluation systems for the perinatal and neonatal periods (including laboratory confirmation and age-stratified reporting) to assess real-world effectiveness and safety (33). Additionally, mother-infant paired serological follow-up should be incorporated into program design to evaluate neonatal antibody titers at birth, antibody waning over time, and the so-called “blunting” effect on infants’ active immunity. This would also involve weighing the trade-offs between short-term passive protection and long-term immunological impacts (34).

This study also has several limitations. First, our serosurvey is cross-sectional and conducted among sentinel hospitals in one province; while the sample size is sizable (N = 1,205), it may not fully represent all pregnant women in China. Second, the risk model we used is deliberately simple and scenario-based: it decomposes risk into multiplicative components and does not capture the full complexity of transmission dynamics (e.g., age-structured contact patterns at the community level, indirect herd effects of adult vaccination, or temporal clustering). However, the model’s transparency makes its assumptions explicit and facilitates sensitivity analyses across plausible parameter ranges. Third, several key parameters—including true community incidence and mother-to-infant transmission probability—are uncertain and context-dependent; reported surveillance incidence likely underestimates true infection incidence because pertussis is often underdiagnosed in adults. Hence, we present a broad range of scenarios to reflect this uncertainty and demonstrate how the absolute impact depends on these inputs. Finally, we assumed a counterfactual maternal protection level of *p*_m_=80.0% achievable with high-coverage Tdap programs; however, the actual seroprotection achieved and program effectiveness in China would require local evaluation.

In conclusion, by integrating serological evidence from pregnant women with scenario-based risk estimation, this study identifies the existing pertussis immunity gap among childbearing-age women in China and its potential threat to newborns under varying epidemiological scenarios. Our quantitative analysis provides empirical evidence to inform the evaluation of introducing maternal Tdap vaccination during pregnancy. We recommend that policymakers, when considering the implementation of this strategy, concurrently strengthen surveillance and evaluation mechanisms to ensure the policy is implemented safely, effectively, and cost-effectively.

## Data Availability

The original contributions presented in the study are included in the article/**Supplementary Material**. Further inquiries can be directed to the corresponding authors.
