# Supplementary material for: Low maternal pertussis protection and quantified infant risk: supporting maternal Tdap vaccination in China
Source: Front Immunol. 2026 Jan 2;16:1687663. doi: 10.3389/fimmu.2025.1687663 (PMC12808340; doi:10.3389/fimmu.2025.1687663)
Supplement: Supplementary file 1 [file Table1.docx]

Table S1. Projected National Annual Infant Pertussis Cases (0–6 Months) and Averted Cases via Maternal Tdap Vaccination Under Varied Incidence and Coverage Scenarios in China

| ***I_w_* per 100k PY** | **Current (*p_m_*=0.0357) cases /100k births** | **National annual cases (9.54M births)** | **Partial coverage (*****p_m_*=0.50) national cases** | **High coverage (*****p_m_*=0.80) national cases** | **Annual cases averted (partial)** | **Annual cases averted (high)** |
| --- | --- | --- | --- | --- | --- | --- |
| 2.15 | 0.2073 | 19.8 | 10.3 | 4.1 | 9.5 | 15.7 |
| 10 | 0.9643 | 92.0 | 47.7 | 19.1 | 44.3 | 72.0 |
| 50 | 4.822 | 459.9 | 238.5 | 95.4 | 221.4 | 364.5 |
| 100 | 9.643 | 919.9 | 477.0 | 190.8 | 442.9 | 729.1 |
| 500 | 48.216 | 4,599.7 | 2,385.0 | 954.0 | 2,214.7 | 3,645.7 |
